# Delay in booster schedule as a control parameter in vaccination dynamics

**DOI:** 10.1007/s00285-019-01424-6

**Published:** 2019-09-07

**Authors:** Zhen Wang, Gergely Röst, Seyed M. Moghadas

**Affiliations:** 1 Agent-Based Modelling Laboratory, York University, Toronto, M3J 1P3 Canada; 2 Mathematical Institute, University of Oxford, Woodstock Road, Oxford, OX2 6GG UK; 3 Bolyai Institute, University of Szeged, Aradi vértanúk tere 1, Szeged, 6720 Hungary

**Keywords:** Vaccination, Booster schedule, Delay equations, Reproduction number, Persistence, Primary 92D30, Secondary 93C23, 34K05, 37M05

## Abstract

The use of multiple vaccine doses has proven to be essential in providing high levels of protection against a number of vaccine-preventable diseases at the individual level. However, the effectiveness of vaccination at the population level depends on several key factors, including the dose-dependent protection efficacy of vaccine, coverage of primary and booster doses, and in particular, the timing of a booster dose. For vaccines that provide transient protection, the optimal scheduling of a booster dose remains an important component of immunization programs and could significantly affect the long-term disease dynamics. In this study, we developed a vaccination model as a system of delay differential equations to investigate the effect of booster schedule using a control parameter represented by a fixed time-delay. By exploring the stability analysis of the model based on its reproduction number, we show the disease persistence in scenarios where the booster dose is sub-optimally scheduled. The findings indicate that, depending on the protection efficacy of primary vaccine series and the coverage of booster vaccination, the time-delay in a booster schedule can be a determining factor in disease persistence or elimination. We present model results with simulations for a vaccine-preventable bacterial disease, *Heamophilus influenzae* serotype b, using parameter estimates from the previous literature. Our study highlights the importance of timelines for multiple-dose vaccination in order to enhance the population-wide benefits of herd immunity.

## Introduction

Vaccination remains the most effective intervention measure in preventing many infectious diseases (Ehreth 2003). Conferring high levels of protection against a number of vaccine-preventable diseases requires more than one dose of vaccine that may be offered at different ages according to specific schedules set by vaccination programs (Jackson et al. 2012; Riolo et al. 2013; Riolo and Rohani 2015). For instance, vaccine schedules against *Haemophilus influenzae* serotype b (Hib) recommended for infants includes either 3 primary doses without a booster, or 2 to 3 primary doses plus a booster given at least 6 months after completing the primary series (World Health Organization et al. 2016). However, even in the presence of booster doses, resurgence and outbreaks of some vaccine-preventable diseases still occur, notwithstanding substantial levels of routine primary vaccine series (Jackson et al. 2012; Riolo et al. 2013; Riolo and Rohani 2015). Reduced effectiveness of vaccination has been explicated for the occurrence of such outbreaks due to factors associated with incomplete protection efficacy of primary vaccine series, inadequate coverage of booster doses, waning immunity over time, and the duration of vaccine-induced protection that may be significantly shorter than the average lifetime of the population (Alexander et al. 2006; Riolo and Rohani 2015).

While the importance of age-at-vaccination and booster doses has been documented, the optimal vaccine schedules remains unclear for several vaccine-preventable diseases and scheduling is mainly determined based on epidemiological context in individual settings (Low et al. 2013; Jackson et al. 2013; Riolo and Rohani 2015). The diversity of booster dose schedules observed in immunization programs worldwide could have a significant impact on disease elimination, since the scheduling may also affect the uptake rates of booster vaccination (Fitzwater et al. 2010). This poses a particular challenge for public health immunization programs in the context of deferral and subsequent refusal of booster doses that diminish the herd immunity (Omer et al. 2009; Dubé et al. 2013; Briere et al. 2014), and could lead to disease resurgence. Identification of the optimal booster schedule is therefore an important component of vaccination policies.

Despite the importance of dosing interval between primary and booster vaccination, a theoretical framework to investigate the impact of such interval and varying vaccination schedules on disease dynamics in the population is currently lacking. In this study, we aimed to establish this framework by developing a vaccination model, represented by a system of delay differential equations that describe the dynamics of disease transmission. Using this system, we evaluated the effect of delay in booster dose after primary vaccination on the long-term disease prevalence. We incorporated a number of key parameters into the model including the protection efficacy of primary vaccination, duration of vaccine-induced protection, and the coverages of primary and booster vaccination. We considered the delay as a control parameter, and analyzed the transient and steady-state behaviours of the system, in addition to determining the effect of time interval between primary and booster doses on disease elimination and persistence. We show, by means of simulations, that the threshold of disease control depends critically on the parameter of delay in booster dose for a given protection efficacy of primary vaccination.

To study the dynamics of our vaccination model, we first propose a basic framework without vaccination, derive the basic reproduction number (${\mathcal {R}}_0$), and prove a threshold result for disease elimination in terms of ${\mathcal {R}}_0$. We then construct the general model by incorporating primary and booster vaccination into the basic framework, and analyze its behaviour. Stability of the disease-free equilibrium is investigated, and represented in terms of the control reproduction number (${\mathcal {R}}_c$). When ${\mathcal {R}}_c > 1$, we show the uniform persistence, indicating that the disease elimination is infeasible. Finally, we use parameter values estimated for a bacterial disease, *Heamophilus influenzae* serotype b (Hib), and perform simulations to illustrate the model results by varying time interval between primary and booster doses, represented by the delay parameter.

## The basic framework

**Fig. 1:**
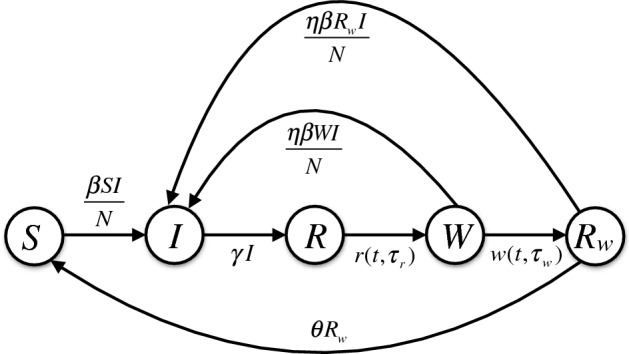
Schematic diagram for the basic structure of the model in the absence of vaccination

To develop the basic framework, we divided a population of constant size *N* into several compartments to represent the epidemiological statuses of individuals as susceptible (*S*), infectious (*I*), recovered and fully protected (*R*), and partially protected (*W*, and $R_w$). The distinction between the two classes *W* and $R_w$ is based on the consideration that partial protection following natural infection may last for a certain period of time (on average) before declining towards negligible levels. We assumed a fixed duration of full protection following recovery from infection. Once this period has elapsed, individuals will have only partial protection, and may become infected at a reduced rate compared with fully susceptible individuals. The duration of partial protection is divided into a period of fixed length (for those in the *W* class), followed by an exponentially distributed period of waning immunity (for those in the $R_w$ class) leading to full susceptibility. Due to the fixed periods of full and partial protection, the dynamics of disease transmission can be expressed by the following set of equations:

1 $$\begin{aligned} S'(t)&=\mu N-\frac{\beta SI}{N}-\mu S + \theta R_w,\nonumber \\ I'(t)&=\frac{\beta SI}{N}+\frac{\eta \beta WI}{N}+\frac{\eta \beta R_w I}{N} -\gamma I -\mu I,\nonumber \\ R(t)&=\int _0^{\tau _r} r(t, a) da, \nonumber \\ W(t)&=\int _0^{\tau _w} w(t, a) da, \nonumber \\ R_w'(t)&= w(t,\tau _w) -\frac{\eta \beta R_w I}{N}-\mu R_w-\theta R_w, \end{aligned}$$

where $\beta$ is the baseline transmission rate; $\gamma$ is the recovery rate of infectious individuals, $\eta$ is the reduction of susceptibility to infection due to partial protection, $\mu$ is the natural death rate (assumed to be the same as the birth rate), $\tau _r$ represents the fixed duration of full protection, $\tau _w$ represents the fixed duration of partial protection; and $\theta$ is the rate of loss of immunity in partially protected individuals in the $R_w$ class. A schematic diagram of the transitions between different classes of individuals in this basic framework is represented in Fig. 1.

Let *a*, referred to as ‘age’, be the time elapsed since individuals enter each class. Thus, *r*(*t*, *a*) and *w*(*t*, *a*) represent the density, with respect to age *a* at time *t*, of recovered individuals having full protection, and those having partial protection during the fixed period before moving to the exponentially distributed period, respectively. Using the above notation, we have the following equations:

2 $$\begin{aligned} \begin{aligned} \left( \frac{\partial }{\partial t}+\frac{\partial }{\partial a} \right) r(t,a)&=-\mu r(t,a),\\ r(t, 0)&= \gamma I(t),\\ \left( \frac{\partial }{\partial t}+\frac{\partial }{\partial a} \right) w(t,a)&=-\mu w(t,a)-\frac{\eta \beta I(t)}{N}w(t,a),\\ w(t, 0)&= r(t, \tau _r). \end{aligned} \end{aligned}$$

Solving along the characteristics gives:

3 $$\begin{aligned} \begin{aligned} r(t,\tau _r)&= r(t-\tau _r,0)e^{-\mu \tau _r}=\gamma I(t-\tau _r)e^{-\mu \tau _r},\\ w(t,\tau _w)&= w(t-\tau _w,0)e^{-\mu \tau _w-\eta {\hat{\beta }}\displaystyle \int _{t-\tau _w}^tI(u) du}\\&= r(t-\tau _w, \tau _r)e^{-\mu \tau _w-\eta {\hat{\beta }}\displaystyle \int _{t-\tau _w}^tI(u) du}\\&=\gamma I(t-\tau _r-\tau _w)e^{-\mu (\tau _r+\tau _w)-{\eta {{\hat{\beta }}}}\displaystyle \int _{t-\tau _w}^{t}I(u) du}, \end{aligned} \end{aligned}$$

where, for simplicity we used the notation ${{\hat{\beta }}}= \beta /N$. Note that system (1) includes integral and differential equations. By differentiating the *R* and *W* equations in (1) with respect to *t*, we obtain the following model, which together with (3), constitutes a closed system of delay differential equations:

4 $$\begin{aligned} \begin{aligned} S'(t)&=\mu N-{\hat{\beta }} SI-\mu S + \theta R_w,\\ I'(t)&={\hat{\beta }} SI+\eta {\hat{\beta }} WI+\eta {\hat{\beta }} R_w I -\gamma I -\mu I,\\ R'(t)&=\gamma I -\mu R - r(t,\tau _r),\\ W'(t)&=r(t,\tau _r)-\eta {\hat{\beta }} WI-\mu W - w(t,\tau _w),\\ R_w'(t)&= w(t,\tau _w) -\eta {\hat{\beta }} R_w I-\mu R_w-\theta R_w. \end{aligned} \end{aligned}$$

One can easily check that the population $N(t) = S(t) + I(t) + R(t) + W(t) + R_w(t)$ is indeed constant.

Let $\tau = \tau _r + \tau _w$, and denote by *C* the Banach space $C([-\tau , 0], {\mathbb {R}}^5)$ of continuous functions mapping the interval $[-\tau , 0]$ into ${\mathbb {R}}^5$ equipped with the norm:

$$\begin{aligned} \Vert \phi \Vert =\sup _{\theta \in [-\tau ,0]} |\phi (\theta )|, \end{aligned}$$

where $\phi \in C$ and $|\cdot |$ is a norm in ${\mathbb {R}}^5$. For a continuous function $u: [-\tau , \sigma _\phi )\rightarrow {\mathbb {R}}^5$ with $\sigma _\phi >0$, we define $u_t \in C$ for each $t\ge 0$ by $u_t(\theta ) = u(t + \theta )$, for all $\theta \in [-\tau , 0]$. We choose the initial conditions for system (4) from the set ${\varOmega }\subseteq C$, defined by:

5 $$\begin{aligned} \begin{aligned} {\varOmega }&= \Big \{\phi \in C: \phi _i(s) \ge 0,\ s \in [-\tau , 0], \,\, 1\le i \le 5, \\&\quad \phi _3(0) = \int _{0}^{\tau _r} \gamma e^{-\mu a} \phi _2(-a) da, \\&\quad \phi _4(0) = \int _0^{\tau _w} \gamma e^{-\mu (\tau _r + a)-\eta {\hat{\beta }}\int _{-a}^{0}\phi _2(u) du} \phi _2(-\tau _r-a) da \Big \}. \end{aligned} \end{aligned}$$

The following result shows that system (4) is well-posed in ${\varOmega }$, and the solution semiflow admits a global attractor on ${\varOmega }$.

### Theorem 2.1

For any $\phi \in {\varOmega }$, system (4) has a unique non-negative solution $u(t, \phi )$ satisfying $u_0 = \phi$ and $u_t \in {\varOmega }$ for all $t>0$, and the solution semiflow ${\varPhi }(t)=u_t(\cdot ): {\varOmega }\rightarrow {\varOmega }$ has a compact global attractor. Moreover, the solutions of system (4) with initial conditions in ${\varOmega }$ satisfy the integro-differential equations system (1).

### Proof

We start with the last assertion. From the third equation of (4), we have:

$$\begin{aligned} e^{\mu t} (R^\prime (t) + \mu R(t)) = \gamma e^{\mu t} (I(t) - I(t - \tau _r)e^{-\mu \tau _r}). \end{aligned}$$

Integrating both sides yields:

$$\begin{aligned} \begin{aligned} e^{\mu t}R(t) - R(0)&= \gamma \left( \int _0^t e^{\mu s} I(s) ds - \int _0^t e^{\mu s} I(s-\tau _r) e^{-\mu \tau _r}ds \right) \\&= \gamma \left( \int _0^t e^{\mu s} I(s) ds - \int _{-\tau _r}^{t-\tau _r} e^{\mu s} I(s)ds \right) \\&= \gamma \int _{t-\tau _r}^{t} e^{\mu s} I(s)ds -\gamma \int _{-\tau _r}^{0} e^{\mu s} I(s)ds. \end{aligned} \end{aligned}$$

Therefore, with $R(0 ) = \gamma \int _{-\tau _r}^{0} e^{\mu s} I(s)ds = \gamma \int ^{\tau _r}_{0} e^{-\mu a} I(-a)da$, we have:

6 $$\begin{aligned} R(t) = \gamma \int _{t-\tau _r}^{t} e^{\mu s} I(s)ds = \gamma \int ^{\tau _r}_{0} e^{-\mu a} I(t-a)da=\int _0^{\tau _r} r(t,a)da. \end{aligned}$$

Similarly, by integrating

$$\begin{aligned} e^{\mu t+\eta {\hat{\beta }} \int _{0}^{t} I(u) du} \left( W^\prime (t) + \mu W(t) + \eta {\hat{\beta }} I(t)W(t) \right) , \end{aligned}$$

and using the initial condition $W(0) = \int _{0}^{\tau _w} \gamma e^{-(\tau _r+a) - \eta {\hat{\beta }}\int _{-a}^{0}I(u)du} I(-\tau _r-a) da$, the differential equation $W^\prime (t)$ in (4) gives:

7 $$\begin{aligned} W(t) \!= \!\gamma \int ^{\tau _w}_{0} \gamma I(t-\tau _r\!-\!a)e^{-\mu (\tau _r\!+\!a)\!-\! {\eta {{\hat{\beta }}}}\displaystyle \int _{t-a}^{t}I(u) du} da=\int _0^{\tau _w} w(t,a)da.\qquad \end{aligned}$$

For a given $\phi \in C$, we define

$$\begin{aligned} G(\phi ): = (G_1(\phi ), G_2(\phi ), G_3(\phi ), G_4(\phi ), G_5(\phi )), \end{aligned}$$

with

$$\begin{aligned} \begin{aligned} G_1(\phi )&= \mu N-{\hat{\beta }} \phi _1(0)\phi _2(0)-\mu \phi _1(0) + \theta \phi _5(0),\\ G_2(\phi )&={\hat{\beta }} \phi _1(0)\phi _2(0) + \eta {\hat{\beta }} \phi _2(0)\phi _4(0)+\eta {\hat{\beta }} \phi _2(0)\phi _5(0) -(\gamma +\mu ) \phi _2(0),\\ G_3(\phi )&=\gamma \phi _2(0) -\mu \phi _3(0) - \gamma \phi _2(-\tau _r)e^{-\mu \tau _r},\\ G_4(\phi )&= \gamma \phi _2(-\tau _r)e^{-\mu \tau _r} -\eta {\hat{\beta }} \phi _2(0)\phi _4(0) - \mu \phi _4(0)\\&\quad -\gamma \phi _2(-\tau _r-\tau _w)e^{-\mu (\tau _r+\tau _w)- \eta {\hat{\beta }}\int _{t-\tau _w}^t \phi _2(s) ds},\\ G_5(\phi )&= \gamma \phi _2(-\tau _r-\tau _w)e^{-\mu (\tau _r+\tau _w)-\eta {\hat{\beta }}\int _{t-\tau _w}^t \phi _2(s) ds} - \eta {\hat{\beta }} \phi _2(0)\phi _5(0)\\&\quad - (\mu +\theta ) \phi _5(0). \end{aligned} \end{aligned}$$

Thus, system (4) can be written as $u'(t)=G(u_t)$. We show that $G(\phi )$ is Lipschitzian in $\phi$ within each compact set in *C*, that is for all $M>0$ there is a $K>0$ such that for all $\phi ,\psi \in C$ with $\Vert \phi \Vert \le M$ and $\Vert \psi \Vert \le M$, the inequality $|G(\phi )-G(\psi )|\le K \Vert \phi -\psi \Vert$ holds. We note that there are terms of linear, quadratic and exponential types in *G*. Quadratic terms are all Lipschitzian, and one can see that:

$$\begin{aligned} |\phi _1(0)\phi _2(0)-\psi _1(0)\psi _2(0)|\le & {} |\phi _1(0)\phi _2(0)-\phi _1(0)\psi _2(0)|\\&+|\phi _1(0)\psi _2(0)-\psi _1(0)\psi _2(0)| \\\le & {} 2M \Vert \phi -\psi \Vert . \end{aligned}$$

The Lipschitzian property for the most involved term can also be seen from:

$$\begin{aligned}&|\phi _2(-\tau )e^{-\eta {{\hat{\beta }}} \int _{t-\tau _w}^t \phi _2(s) ds} -\psi _2(-\tau )e^{-\eta {{\hat{\beta }}} \int _{t-\tau _w}^t \psi _2(s) ds}| \\&\quad \le |\phi _2(-\tau )e^{-\eta {{\hat{\beta }}} \int _{t-\tau _w}^t \phi _2(s) ds} -\phi _2(-\tau )e^{-\eta {{\hat{\beta }}} \int _{t-\tau _w}^t \psi _2(s) ds}| \\&\qquad +|\phi _2(-\tau )e^{-\eta {{\hat{\beta }}} \int _{t-\tau _w}^t \psi _2(s) ds} -\psi _2(-\tau )e^{-\eta {{\hat{\beta }}} \int _{t-\tau _w}^t \psi _2(s) ds}| \\&\quad \le M e^{\eta {{\hat{\beta }}} \tau _w M } \eta {{\hat{\beta }}} \tau _w \Vert \phi -\psi \Vert +e^{\eta {{\hat{\beta }}} \tau _w M }\Vert \phi -\psi \Vert , \end{aligned}$$

where we used the mean value theorem $e^x-e^y=e^\xi (x-y)$. Hence, there is a unique solution of the system through $(0, \phi )$ on its maximal interval of existence $[0, \sigma _\phi )$. We note from (4) that any solution satisfies:

8 $$\begin{aligned} I(t)=I(0)e^{\int _0^t {{\hat{\beta }}} S(u)+\eta {{\hat{\beta }}} W(u)+ \eta \hat{\beta }R_w(u) -\gamma -\mu du} , \end{aligned}$$

and hence if $I(0)\ge 0$, then $I(t)\ge 0$ for all $t \in (0,\sigma _\phi )$. From (6) and (7), we find that *R* and *W* are non-negative. Then the non-negativity of *S* and $R_w$ follows from the inequalities

$$\begin{aligned} S'(t) \ge \mu N-{{{\hat{\beta }}} SI}-\mu S, \end{aligned}$$

and

$$\begin{aligned} R_w'(t)\ge -\eta {{\hat{\beta }}} R_w I-\mu R_w-\theta R_w. \end{aligned}$$

The non-negativity and relations (6) and (7) ensure that ${\varOmega }$ is forward invariant. Since the total population is constant, it follows that *S*(*t*) and *I*(*t*) are bounded by *N* and the solutions exist globally. Therefore, the solution semiflow ${\varPhi }(t) = u_t(\cdot ): {\varOmega }\rightarrow {\varOmega }$ is point dissipative. By Theorem 3.6.1 in Hale (1977), ${\varPhi }(t)$ is compact for any $t > \tau$. Thus, from Theorem 3.4.8 in Hale (1988), it follows that ${\varPhi }(t)$ has a compact global attractor in ${\varOmega }$. $\square$

### Basic reproduction number

The basic reproduction number (${\mathcal {R}}_0$) is the average number of new infected individuals generated by a single infected individual introduced into an entirely susceptible population, during the course of infection. According to the theory of epidemics, we expect that the disease will vanish if ${\mathcal {R}}_0 < 1$, while it will persist in the population if ${\mathcal {R}}_0 >1$. New infections occur only in the *S* class with the rate ${{\hat{\beta }}} I$. In a fully susceptible population, $S/N \approx 1$ and the average length of infection is $(\mu +\gamma )^{-1}$, and therefore we define the basic reproduction number as ${\mathcal {R}}_0 = \beta /(\gamma + \mu )$. We proceed by presenting the threshold dynamics for system (4), which determines whether the disease dies out or persists.

### Threshold dynamics

It is clear that system (4) has a unique disease-free equilibrium $E_0 = (N, 0, 0, 0, 0)$. We first show that $E_0$ is globally asymptotically stable when ${\mathcal {R}}_0 < 1$. Then we establish the uniform persistence of the disease when ${\mathcal {R}}_0 >1$ using techniques of persistence theory (Smith and Thieme 2011).

#### Theorem 2.2

If ${\mathcal {R}}_0 < 1$, then the disease-free equilibrium $E_0$ of system (4) is globally asymptotically stable in ${\varOmega }$.

#### Proof

Linearizing system (4) at $E_0$, we obtain the following system:

9 $$\begin{aligned} u^\prime (t) = A_1 u(t) + A_2u(t-\tau _r) + A_3 u(t - \tau _r - \tau _w), \end{aligned}$$

where $$u(t) = (S(t), I(t), R(t), W(t), R_w(t))^T$$, and

$$\begin{aligned} A_1 = \begin{pmatrix} -\mu &{}\quad -\beta &{}\quad 0 &{}\quad 0 &{} \quad \theta \\ 0 &{}\quad \beta -\gamma -\mu &{}\quad 0 &{}\quad 0&{}\quad 0 \\ 0 &{}\quad \gamma &{}\quad -\mu &{}\quad 0 &{}\quad 0 \\ 0 &{}\quad 0 &{}\quad 0 &{}\quad -\mu &{} \quad 0 \\ 0 &{}\quad 0 &{}\quad 0 &{}\quad 0 &{}\quad -(\mu + \theta ) \end{pmatrix}, \end{aligned}$$

$A_2=(A_2)_{ij}$, $1\le i, j \le 5$, with $(A_2)_{32}= -\gamma e^{-\mu \tau _r}$, $(A_2)_{42}= \gamma e^{-\mu \tau _r}$ and all other components are zero; $A_3=(A_3)_{ij}$, $1\le i, j \le 5$, with $(A_3)_{42}= -\gamma e^{-\mu (\tau _r+ \tau _w)}$, $(A_3)_{52}= \gamma e^{-\mu (\tau _r+ \tau _w)}$ and all other components are zero. The characteristic equation of system (9) has the form:

$$\begin{aligned} \det (\lambda {\mathbb {I}} - A_1 - e^{-\tau _r\lambda }A_2 - e^{-(\tau _r+\tau _w)\lambda }A_3)&= 0, \end{aligned}$$

which gives $(\lambda +\mu )^3(\lambda +\mu +\theta )(\lambda -\beta +\gamma +\mu ) = 0$. Since ${\mathcal {R}}_0 < 1$, we have $\beta - (\gamma + \mu ) < 0$, which implies that $E_0$ is asymptotically stable.

Now we prove that $E_0$ is globally attractive in ${\varOmega }$. We consider the following inequality:

$$\begin{aligned} \begin{aligned} I^\prime (t)&\le {\hat{\beta }} (S + W + R_w) I - (\gamma + \mu )I \le ({\mathcal {R}}_0-1)(\gamma + \mu )I. \end{aligned} \end{aligned}$$

Thus, $I(t)\le I(0)e^{({\mathcal {R}}_0-1)(\gamma + \mu )t}$, and hence $I(t)\rightarrow 0$ as $t \rightarrow \infty$. From (6) and (7), we find that *R*(*t*) and *W*(*t*) are also converging to zero. For any $\epsilon >0$, and a sufficiently large $t>0$, we have $w(t,\tau _w)<\epsilon$, and the following inequality holds:

$$\begin{aligned} R_w'(t) \le \epsilon - (\mu +\theta )R_w. \end{aligned}$$

This means that $\displaystyle \limsup _{t\rightarrow \infty } R_w(t) \le \frac{\epsilon }{\mu +\theta }$, and therefore $R_w \rightarrow 0$ as $t \rightarrow \infty$. Since the total population is constant, we obtain that $S(t) \rightarrow N$ as $t \rightarrow \infty$. Thus, $\displaystyle \lim _{t\rightarrow \infty } u(t, \phi ) = (N, 0, 0 , 0, 0)$, and we conclude that $E_0$ is globally asymptotically stable. $\square$

We now prove the persistence of disease for ${\mathcal {R}}_0>1$. Considering the semiflow ${\varPhi }$ on ${\varOmega }$, we define the persistence function by:

$$\begin{aligned} P: {\varOmega }\rightarrow {\mathbb {R}}_{+}, \quad P(\phi )=\phi _2(0). \end{aligned}$$

Let

$$\begin{aligned} {\varOmega }_{+}&:=\{\phi \in {\varOmega }|P(\phi )>0\},\\ {\varOmega }_{0}&:=\{\phi \in {\varOmega }|P(\phi )=0\}= {\varOmega }\setminus { {\varOmega }_{+}}, \end{aligned}$$

where ${\varOmega }_{0}$ is called the extinction space corresponding to *P* (that is the collection of states where the disease is not present). From the relation (8), it follows that the sets ${\varOmega }_{0}$ and ${\varOmega }_{+}$ are forward invariant under the semiflow ${\varPhi }$. We now introduce some terminology from persistence theory (Smith and Thieme 2011, Chapters 3.1 and 8.3).

#### Definition 2.3

Let *X* be a nonempty set and $P: X\rightarrow {\mathbb {R}}_{+}$.

1. A semiflow ${\varPhi }:{\mathbb {R}}_{+}\times X \rightarrow X$ is called uniformly weakly *P*-persistent, if there exists some $\epsilon >0$ such that
  $$\begin{aligned} \limsup _{t\rightarrow \infty } P({\varPhi }(t,x))>\epsilon \qquad \forall \,x\in X,~ P(x)>0. \end{aligned}$$
2. A semiflow ${\varPhi }$ is called uniformly (strongly) *P*-persistent, if there exists some $\epsilon >0$ such that
  $$\begin{aligned} \liminf _{t\rightarrow \infty } P({\varPhi }(t,x))>\epsilon \qquad \forall \,x\in X,~ P(x)>0. \end{aligned}$$
3. A set $M\subseteq X$ is called weakly *P*-repelling if there is no $x\in X$ such that $P(x)>0$ and ${\varPhi }(t,x)\rightarrow M$ as $t\rightarrow \infty$.

#### Theorem 2.4

If ${\mathcal {R}}_0>1$, then the semiflow ${\varPhi }$ is uniformly *P*-persistent, i.e., there is a $\delta >0$ such that for any solution $\liminf _{t \rightarrow \infty } I(t)\ge \delta$.

#### Proof

First we show that $E_0$ is weakly *P*-repelling. Suppose that there exists $\psi _{0}\in {\varOmega }$ such that $P(\psi _{0})>0$ with

10 $$\begin{aligned} \lim _{t\rightarrow \infty }{\varPhi }(t,\psi _{0})=E_0. \end{aligned}$$

For such a solution, $I(0)>0$ and $\lim _{t \rightarrow \infty } I(t)=0$. Let $\epsilon >0$ small so that ${\mathcal {R}}_0(1-\frac{\epsilon }{N})>1$. Thus, for sufficiently large *t* we have $\Vert {\varPhi }(t,\psi _{0})-E_0\Vert <\epsilon$, and

$$\begin{aligned} \begin{aligned} I^\prime&\ge {\hat{\beta }} S I - (\gamma +\mu )I \ge \frac{\beta (N-\epsilon )}{N}I - (\gamma +\mu )I\\&=\left( {\mathcal {R}}_0(1-\frac{\epsilon }{N})-1\right) (\gamma +\mu )I>0, \end{aligned} \end{aligned}$$

which contradicts the convergence of *I* to zero. Hence, $E_0$ is weakly *P*-repelling. Notice that whenever $I(t)\equiv 0$, from (6) and (7) we have $R(t)\equiv 0$ , $W(t)\equiv 0$, and $w(t,\tau _w)\equiv 0$, and consequently $R_w \rightarrow 0$ and $S \rightarrow N$ as $t\rightarrow \infty$. Therefore $\displaystyle \cup _{\phi \in {\varOmega }_{0}}\omega (\phi )=\{E_0\}$, and one can see from Theorem 8.17 in Smith and Thieme (2011) that ${\varPhi }$ is uniformly weakly *P*-persistent. Since ${\varPhi }$ has a compact global attractor on ${\varOmega }$, we can apply Theorem 4.5 in Smith and Thieme (2011) to conclude that ${\varPhi }$ is uniformly *P*-persistent. $\square$

Theorems 2.2 and 2.4 indicate that the disease dynamics are completely determined by the basic reproduction number ${\mathcal {R}}_0$. In the following, we extend our model by introducing the primary and booster vaccination, and analyze the persistence dynamics of the resulting system.

**Table 1:** Description of vaccine-related individual classes in the vaccination model

Variable	Description
$V_s$	Newborns who will receive primary vaccine in $\tau _s$ period of time following birth
$V_p$	Primary vaccinated individuals who may receive booster dose
$V_d$	Partially protected individuals who will not receive booster dose
$V_w$	Primary vaccinated individuals in whom vaccine-induced protection wanes over
$V_b$	Individuals who have received booster vaccination and are currently fully protected

## The general vaccination model

**Fig. 2:**
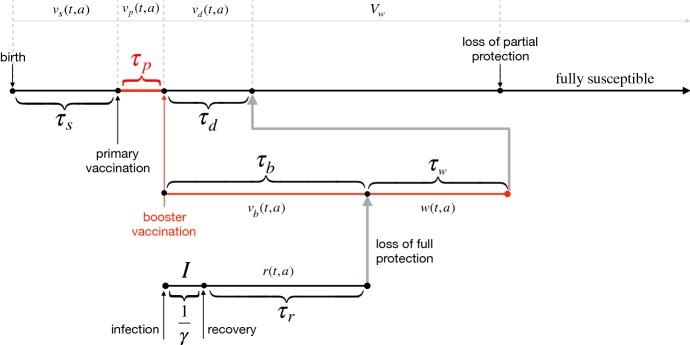
Schematic diagram for timelines of primary and booster vaccination with delay, and durations of vaccine-induced and naturally acquired protection

The vaccination model includes additional classes of individuals who are vaccinated with primary series; partially protected following primary vaccination; and fully protected following booster vaccination (See Table 1). A schematic diagram for timelines of primary and booster vaccination with delays is represented in Fig. 2. We assume that a fraction *p* of newborns will receive primary vaccine series in the first $\tau _s$ period of their life. The remaining fraction of newborns will be recruited to the susceptible class and can therefore become infected through contacts with infectious individuals. The primary vaccination is assumed to provide partial protection for a certain period of time during which infection can occur with a lower rate compared with fully susceptible individuals. We also assume that partial protection induced by primary vaccine gradually wanes over time, and individuals who forgo booster vaccination will eventually become susceptible. Similar to the basic framework, we consider a fixed duration of partial protection after primary vaccination, followed by an exponentially distributed period of waning immunity leading to full susceptibility. Those who have received primary vaccination may also receive booster dose. We assume that, similar to recovery from infection, booster vaccination provides a fixed duration of full protection, followed by fixed and exponentially distributed durations of partial protection.

In order to mathematically express the model, we let *a* represent the age since the individuals enter each class, and define $v_s(t, a)$, $v_p(t,a)$, and $v_d(t,a)$ to represent the density, with respect to age *a* at current time *t*, of primary vaccinated individuals, those who are partially protected as a result of primary vaccination and eligible to receive booster dose, and those who are partially protected by primary vaccination and will not receive booster dose, respectively. Let $\tau _p$ ($0\le \tau _p\le \tau _w$) represent the delay in receiving booster vaccination within the fixed period of partial protection following primary vaccination. For those who will not receive the booster, we denote by $\tau _d$ the remaining period of time within the fixed duration of partial protection, i.e., $\tau _p+\tau _d=\tau _w$. Description of all model parameters are provided in Table 2.

**Table 2:** Description of the model parameters and their associated values (ranges) extracted from the published literature

Parameter	Description	Value (range)
${\mathcal {R}}_0$	Basic reproduction number	1.2 (1.1–1.4)
$\mu$	Birth and natural death rate	1/70 per year
$\gamma$	Recovery rate of infection	7.3 per year
$\beta ={\mathcal {R}}_0(\gamma +\mu )$	baseline transmission rate of infection	8.777 per year
*p*	Coverage of primary vaccination	0.9 (0–1)
$\rho$	Coverage of booster vaccination	Variable (0–1)
$\eta$	Reduced susceptibility during partial protection	Variable (0–1)
$\tau _s$	Age at primary vaccination	6 months
$\tau _p$	Delay in booster vaccination	Variable (0–4) years
$\tau _w$	Fixed period of partial protection	4 years
$\tau _b$	Fixed period of full protection following booster	6 years
$\tau _r$	Fixed period of full protection following recovery	4 years
$\theta$	Rate of loss of partial protection	0.1667 per year

With the above notation, the model can be expressed by the following system of integro-differential equations:

11 $$\begin{aligned} S'(t)&=(1-p)\mu N-{\hat{\beta }} SI-\mu S + \theta V_w,\nonumber \\ V_s(t)&=\int _0^{\tau _s} v_s(t,a)da, \nonumber \\ V_p(t)&=\int _{0}^{\tau _p} v_p(t,a)da,\nonumber \\ V_d(t)&=\int _{0}^{\tau _d} v_d(t,a)da,\nonumber \\ V_w'(t)&=v_d(t,\tau _d) -\eta {\hat{\beta }} V_w I -(\mu +\theta ) V_w +w(t,\tau _w), \nonumber \\ V_b(t)&=\int _{0}^{\tau _b} v_b(t,a)da,\nonumber \\ W(t)&=\int _{0}^{\tau _w} w(t,a)da,\nonumber \\ I'(t)&={\hat{\beta }} I[S+V_s+\eta (V_p+V_d+V_w+W)] -\gamma I -\mu I,\nonumber \\ R(t)&=\int _0^{\tau _r}r(t,a)da, \end{aligned}$$

with population densities expressed by:

12 $$\begin{aligned} \left( \frac{\partial }{\partial t}+\frac{\partial }{\partial a} \right) v_s(t,a)&=-\mu v_s(t,a)-{\hat{\beta }} I(t)v_s(t,a),\nonumber \\ v_s(t,0)&=p\mu N, \end{aligned}$$

13 $$\begin{aligned} \left( \frac{\partial }{\partial t}+\frac{\partial }{\partial a} \right) v_p(t,a)&=-\mu v_p(t,a)-\eta {\hat{\beta }} I(t)v_p(t,a), \nonumber \\ v_p(t,0)&=v_s(t,\tau _s),\end{aligned}$$

14 $$\begin{aligned} \left( \frac{\partial }{\partial t}+\frac{\partial }{\partial a} \right) v_d(t,a)&=-\mu v_d(t,a)-\eta {\hat{\beta }} I(t)v_d(t,a), \nonumber \\ v_d(t,0)&=(1-\rho )v_p(t,\tau _p),\end{aligned}$$

15 $$\begin{aligned} \left( \frac{\partial }{\partial t}+\frac{\partial }{\partial a} \right) v_b(t,a)&=-\mu v_b(t,a), \nonumber \\ v_b(t,0)&=\rho v_p(t,\tau _p),\end{aligned}$$

16 $$\begin{aligned} \left( \frac{\partial }{\partial t}+\frac{\partial }{\partial a} \right) r(t,a)&=-\mu r(t,a), \nonumber \\ r(t,0)&=\gamma I(t),\end{aligned}$$

17 $$\begin{aligned} \left( \frac{\partial }{\partial t}+\frac{\partial }{\partial a} \right) w(t,a)&=-\mu w(t,a)-\eta {\hat{\beta }} I(t)w(t,a), \nonumber \\ w(t,0)&=v_b(t,\tau _b) + r(t,\tau _r). \end{aligned}$$

In this model, we considered a single class $V_w$ for partially protected individuals with exponentially distributed duration of protection, regardless of whether the immunity was conferred by vaccination or natural infection. Thus, the $R_w$ class from the basic framework is included in the $V_w$ class in the vaccination model. Solving along the characteristics gives:

18 $$\begin{aligned} \begin{aligned} v_s(t,\tau _s)&=v_s(t-\tau _s,0)e^{-\mu \tau _s-{\hat{\beta }}{\displaystyle \int _{t-\tau _s}^t}I(u) du}=p\mu N e^{-\mu \tau _s-{\hat{\beta }}{\displaystyle \int _{t-\tau _s}^t}I(u) du},\\ v_p(t,\tau _p)&=v_p(t-\tau _p,0)e^{-\mu \tau _p- \eta {\hat{\beta }}{\displaystyle \int _{t-\tau _p}^t} I(u) du },\\ v_d(t,\tau _d)&=v_d(t-\tau _d,0)e^{-\mu \tau _d-\eta {\hat{\beta }}{\displaystyle \int _{t-\tau _d}^t} I(u)du },\\ v_b(t,\tau _b)&=v_b(t-\tau _b,0)e^{-\mu \tau _b}=\rho v_p(t-\tau _b,\tau _p)e^{-\mu \tau _b},\\ r(t,\tau _r)&=r(t-\tau _r,0)e^{-\mu \tau _r}=\gamma I(t-\tau _r)e^{-\mu \tau _r},\\ w(t,\tau _w)&=w(t-\tau _w,0)e^{-\mu \tau _w-\eta {\hat{\beta }}\displaystyle \int _{t-\tau _w}^tI(u) du}. \end{aligned} \end{aligned}$$

Differentiating the integral equations $V_s$, $V_p$, $V_d$, $V_b$, *W* and *R* in (11) and substituting the density functions, we obtain the following system of differential equations with delays:

19 $$\begin{aligned} S'(t)&=(1-p)\mu N-{\hat{\beta }}SI-\mu S + \theta V_w,\nonumber \\ V_s'(t)&=p\mu N\Big (1-e^{-\mu \tau _s-{\hat{\beta }}A}\Big )-{\hat{\beta }} V_sI-\mu V_s,\nonumber \\ V_p'(t)&=p\mu N \Big (e^{-\mu \tau _s-{\hat{\beta }} A} - e^{-\mu (\tau _s+\tau _p)-\eta {\hat{\beta }}B-{\hat{\beta }}A(t-\tau _p)}\Big ) -\eta {\hat{\beta }} V_p I-\mu V_p, \nonumber \\ V_d'(t)&=(1-\rho )p\mu N e^{-\mu (\tau _s+\tau _p)-\eta {\hat{\beta }}B-{\hat{\beta }}A(t-\tau _p)} \nonumber \\&\quad - (1-\rho )p\mu N e^{-\mu (\tau _s+\tau _p+\tau _d)-\eta {\hat{\beta }}C-{\hat{\beta }}A(t-\tau _w)} -\eta {\hat{\beta }} V_d I-\mu V_d, \nonumber \\ V_w'(t)&=(1-\rho )p\mu N e^{-\mu (\tau _s+\tau _p+\tau _d)-\eta {\hat{\beta }}C-{\hat{\beta }}A(t-\tau _w)} -\eta {\hat{\beta }} V_w I-(\mu +\theta ) V_w\nonumber \\&\quad +\rho p \mu N e^{-\mu (\tau _s+\tau _p+\tau _b+\tau _w)- \eta {\hat{\beta }}C-\eta {\hat{\beta }}B(t-\tau _b-\tau _w)- {\hat{\beta }}A(t-\tau _p-\tau _b-\tau _w)} \nonumber \\&\quad +\gamma I(t-\tau _r-\tau _w)e^{-\mu \tau _r-\mu \tau _w-\eta {{\hat{\beta }}} C},\nonumber \\ V_b^\prime (t)&= \rho p\mu N e^{-\mu (\tau _s+\tau _p)- \eta {\hat{\beta }} B - {\hat{\beta }}A(t-\tau _p)} - \mu V_b\nonumber \\&\quad - \rho p\mu N e^{-\mu (\tau _s+\tau _p+\tau _b) - \eta {\hat{\beta }}B(t-\tau _b)-{\hat{\beta }}A(t-\tau _p-\tau _b)}, \nonumber \\ W'(t)&=\rho p\mu N e^{-\mu (\tau _s+\tau _p+\tau _b)- \eta {\hat{\beta }} B(t-\tau _b)-{\hat{\beta }}A(t-\tau _p-\tau _b)}-\eta {\hat{\beta }}WI-\mu W\nonumber \\&\quad -\rho p\mu N e^{-\mu (\tau _s+\tau _p+\tau _b+\tau _w) -\eta {\hat{\beta }}B(t-\tau _b-\tau _w)-{\hat{\beta }}A (t-\tau _p-\tau _b-\tau _w)-\eta {\hat{\beta }}C}\nonumber \\&\quad -\gamma \bigg (I(t-\tau _r-\tau _w)e^{-\mu \tau _w-\eta {\hat{\beta }}C} - I(t-\tau _r)\bigg )e^{-\mu \tau _r}\nonumber ,\\ I'(t)&={\hat{\beta }}\bigg (S+V_s+\eta (V_p+V_d+V_w+W)\bigg )I -\gamma I -\mu I, \nonumber \\ R^\prime (t)&= \gamma I - \gamma I(t-\tau _r)e^{-\mu \tau _r} - \mu R, \end{aligned}$$

where

$$\begin{aligned} \begin{aligned}&A(t)=\displaystyle \int _{t-\tau _s}^t I(u) du, \quad&B(t)=\displaystyle \int _{t-\tau _p}^t I(u) du, \quad&C(t)=\displaystyle \int _{t-\tau _w}^{t} I(u) du. \end{aligned} \end{aligned}$$

The total population $S(t) + V_s(t) +V_p(t) +V_d(t) +V_w(t) +V_b(t) +W(t) +I(t) +R(t)=N$. Note that the equations of $V_b$ and *R* are decoupled from the rest of the model.

In the following, we show that (19) is well-posed, and further define the disease-free equilibrium and the control reproduction number. Let $\tau _c = \max \{\tau _s + \tau _p+ \tau _b+\tau _w, \ \tau _r+\tau _w \}$. We choose the initial conditions for system (19) from the set ${\mathcal {X}}$, which is defined by

20 $$\begin{aligned} {\mathcal {X}}&= \Big \{\phi \in C([-\tau _c, 0], {\mathbb {R}}^9): \phi _i(s) \ge 0, \ s\in [-\tau _c, 0], \ 1 \le i \le 9, \nonumber \\&\quad \phi _2(0) = \int _{0}^{\tau _s} p\mu N e^{-\mu a-{\hat{\beta }}\int _{-a}^{0} \phi _8(s)ds}da,\nonumber \\&\quad \phi _3(0) =\int _{0}^{\tau _p} p\mu N e^{-\mu (\tau _s + a) - \eta {\hat{\beta }}\int _{-a}^{0}\phi _8(s)ds-{\hat{\beta }}\int _{-\tau _s-a}^{-a}\phi _8(s)ds} da,\nonumber \\&\quad \phi _4(0) = \int _{0}^{\tau _d} (1-\rho ) p\mu N e^{-\mu (\tau _s +\tau _p+ a) - \eta {\hat{\beta }}\int _{-\tau _p-a}^{0}\phi _8(s)ds -{\hat{\beta }}\int _{-\tau _s-\tau _p-a}^{-\tau _p-a}\phi _8(s)ds}da, \nonumber \\&\quad \phi _6(0) = \int _{0}^{\tau _b}\rho p\mu N e^{-\mu (\tau _s +\tau _p+ a) - \eta {\hat{\beta }}\int _{-\tau _p-a}^{-a}\phi _8(s)ds -{\hat{\beta }}\int _{-\tau _s-\tau _p-a}^{-\tau _p-a}\phi _8(s)ds}da, \nonumber \\&\quad \phi _7(0) = \int _{0}^{\tau _w}\rho p\mu N e^{-\mu (\tau _s +\tau _p+ \tau _b+ a) - \eta {\hat{\beta }}\int _{-a}^{0}\phi _8(s)ds - \eta {\hat{\beta }}\int _{-\tau _p-\tau _b-a}^{-\tau _b-a}\phi _8(s)ds}\nonumber \\&\qquad \times e^{-{\hat{\beta }}\int _{-\tau _s-\tau _p-\tau _b-a}^{-\tau _p-\tau _b-a}\phi _8(s)ds} + \gamma \phi _8(-\tau _r-a)e^{-\mu (\tau _r+a) - \eta {\hat{\beta }} \int _{-a}^0 \phi _8(s)ds} da, \nonumber \\&\quad \phi _9(0) = \int _{0}^{\tau _r}\gamma e^{-\mu a}\phi _8(-a) da \Big \}. \end{aligned}$$

### Theorem 3.1

For any $\phi \in {\mathcal {X}}$, system (19) has a unique non-negative solution $U(t, \phi )$ satisfying $U_0 = \phi$ and $U_t \in {\mathcal {X}}$, and the solution semiflow ${\varPhi }(t) = U_t (\cdot ): {\mathcal {X}}\rightarrow {\mathcal {X}}$ has a compact global attractor. Moreover, the solutions of system (19) with initial conditions in ${\mathcal {X}}$ satisfy the integro-differential equations system (11).

### Proof

The proof is similar to Theorem 2.1. $\square$

### Reproduction number

Recall that in the absence of vaccination, system (19) reduces to (4) and the basic reproduction number is given by ${\mathcal {R}}_0=\beta /(\mu +\gamma )$. Letting $I(t) \equiv 0$, we obtain the unique disease-free equilibrium of system (19), $E^*_0 = (S^\circ , V_s^\circ , V_p^\circ , V_d^\circ , V_w^\circ , V_b^\circ , W^\circ , 0, 0)$, where

$$\begin{aligned} \begin{aligned} S^\circ&=(1-p)N + \frac{\theta p N}{\mu +\theta }\Big [(1-\rho )e^{-\mu \tau _s}+\rho e^{-\mu (\tau _s+\tau _p+\tau _b)}\Big ]e^{-\mu \tau _w},\\ V^\circ _s&=pN\big (1-e^{-\mu \tau _s}\big ),\\ V^\circ _p&=pN\big (1-e^{-\mu \tau _p}\big )e^{-\mu \tau _s},\\ V^\circ _d&=(1-\rho )pN\big (1-e^{-\mu \tau _d}\big )e^{-\mu (\tau _s+\tau _p)},\\ V^\circ _w&=\frac{\mu p N}{\mu +\theta }\Big [(1-\rho )e^{-\mu \tau _s}+\rho e^{-\mu (\tau _s+\tau _p+\tau _b)}\Big ]e^{-\mu \tau _w},\\ V^\circ _b&= \rho pN\big (1-e^{-\mu \tau _b}\big )e^{-\mu (\tau _s+\tau _p)},\\ W^\circ&=\rho p N\big (1-e^{-\mu \tau _w}\big )e^{-\mu (\tau _s+\tau _p+\tau _b)}. \end{aligned} \end{aligned}$$

Linearizing system (19) at $E^*_0$, we obtain the following equation for the infection class:

21 $$\begin{aligned} I^\prime (t) = {\hat{\beta }}[S^\circ +V_s^\circ + \eta (V_p^\circ +V_d^\circ +V_w^\circ +W^\circ )]I -(\gamma + \mu ) I. \end{aligned}$$

We now introduce the reproduction number following the idea in (Xu and Zhao 2012). Denote by $x_0$ the number of infectious individuals at time $t = 0$, and $x_1(t)$ be the remaining population at time *t*. Thus,

$$\begin{aligned} x_1(t) = x_0 e^{-(\gamma + \mu ) t}. \end{aligned}$$

Thus, from (21), the total number of newly infected cases is

$$\begin{aligned} {\bar{x}}_1&= {\hat{\beta }}[S^\circ +V_s^\circ +\eta (V_p^\circ + V_d^\circ +V_w^\circ +W^\circ )]\displaystyle \int _{0}^{\infty } x_1(t)dt\\&= \displaystyle \frac{{\hat{\beta }}}{\gamma +\mu }\left( S^\circ +V_s^\circ +\eta (V_p^\circ +V_d^\circ +V_w^\circ +W^\circ )\right) x_0. \end{aligned}$$

Therefore, we define the reproduction number in the presence of vaccination by

$$\begin{aligned} \begin{aligned} {\mathcal {R}}_c&= \frac{{\hat{\beta }}}{\gamma +\mu }\left( S^\circ +V_s^\circ +\eta (V_p^\circ +V_d^\circ +V_w^\circ +W^\circ ) \right) \\&= {\mathcal {R}}_0\Big [(1-pe^{-\mu \tau _s})+p\Big (\frac{\theta + \eta \mu }{\mu +\theta }\Big )\Big ((1-\rho )e^{-\mu \tau _s}+\rho e^{-\mu (\tau _s+\tau _p+\tau _b)}\Big )e^{-\mu \tau _w}\\&\qquad +\eta p\big (1-e^{-\mu \tau _p}\big )e^{-\mu \tau _s} +\eta (1-\rho ) p (1-e^{-\mu \tau _d})e^{-\mu (\tau _s+\tau _p)}\\&\qquad +\eta \rho p\big (1-e^{-\mu \tau _w}\big )e^{-\mu (\tau _s+\tau _p+\tau _b)}\Big ], \end{aligned} \end{aligned}$$

which can be interpreted as the total number of new infections generated by a single infectious individual in all non-infection classes during the average infectious period $1/(\mu +\gamma )$.

### Threshold dynamics

#### Local stability

Here, we show disease elimination for sufficiently small *I* (corresponding to solutions in a small neighbourhood of $E_0^*$) if ${\mathcal {R}}_c < 1$.

Notice that $V_b$ and *R* in system (19) are independent of other state variables. In the following, we consider (19) with the additional equations:

22 $$\begin{aligned} \begin{aligned} A^\prime (t)&= I(t) - I(t-\tau _s), \\ B^\prime (t)&= I(t) - I(t-\tau _p), \\ C^\prime (t)&= I(t)- I(t-\tau _w). \\ \end{aligned} \end{aligned}$$

Linearizing (19) at $E_0^*$, we have

23 $$\begin{aligned} U^\prime (t)= & {} D U(t) + D_1 U(t-\tau _s) + D_2 U(t - \tau _p) + D_3 U(t - \tau _w) + D_4 U(t - \tau _r) \nonumber \\&+ D_5 U(t- \tau _b) + D_6 U(t - (\tau _p + \tau _b)) +D_7 U(t - (\tau _r + \tau _w)) \nonumber \\&+ D_8 U(t - (\tau _b + \tau _w)) +D_9 U(t - (\tau _p + \tau _b + \tau _w)), \end{aligned}$$

where $$U(t) = \left( S(t), V_s(t), V_p(t), V_d(t), V_w(t), W(t), I(t), A(t), B(t), C(t)\right) ^T$$, and

$$\begin{aligned} D = \begin{pmatrix} D_{11} &{} D_* \\ O_{4\times 6} &{} D_{21} \end{pmatrix}. \end{aligned}$$

This is a block triangular matrix with the zero block $O_{4\times 6}$, and

$$\begin{aligned}&D_{11} = \begin{pmatrix} -\mu &{}\quad 0 &{}\quad 0 &{}\quad 0 &{}\quad \theta &{}\quad 0 \\ 0 &{}\quad -\mu &{}\quad 0 &{}\quad 0 &{}\quad 0 &{}\quad 0 \\ 0 &{}\quad 0 &{}\quad -\mu &{}\quad 0 &{}\quad 0 &{}\quad 0 \\ 0 &{}\quad 0 &{}\quad 0 &{}\quad -\mu &{}\quad 0 &{}\quad 0 \\ 0 &{}\quad 0 &{}\quad 0 &{}\quad 0 &{}\quad -(\mu +\theta ) &{}\quad 0 \\ 0 &{}\quad 0 &{}\quad 0 &{}\quad 0 &{}\quad 0 &{}\quad -\mu \end{pmatrix}, \\&\qquad D_{21} = \begin{pmatrix} (\gamma + \mu ) ({\mathcal {R}}_c -1) &{}\quad 0 &{}\quad 0 &{}\quad 0 \\ 1 &{}\quad 0 &{}\quad 0 &{}\quad 0 \\ 1 &{}\quad 0 &{}\quad 0 &{}\quad 0 \\ 1 &{}\quad 0 &{}\quad 0 &{}\quad 0 \\ \end{pmatrix}. \end{aligned}$$

All matrices $D_j$, $j=1,\dots 9$, that appear in (23) can also be derived from (19) and (22).

##### Theorem 3.2

If ${\mathcal {R}}_c < 1$, then the disease-free equilibrium $E_0^*$ of system (19) is locally asymptotically stable.

##### Proof

The characteristic equation of the linearized system at $E_0^*$ is

$$\begin{aligned} \det \left[ D+e^{-\lambda \tau _s} D_1 + \dots + e^{-\lambda (\tau _p+\tau _b+\tau _w)} D_9 - \lambda {\mathbb {I}} \right] =0, \end{aligned}$$

which, after straightforward calculations, simplifies to

$$\begin{aligned} \lambda ^3 (\lambda + \mu )^5 (\lambda + \mu + \theta ) (\lambda - (\gamma +\mu )({\mathcal {R}}_c - 1)) = 0. \end{aligned}$$

Since ${\mathcal {R}}_c < 1$, the local stability of $E_0^*$ is proven. $\square$

##### Remark 3.3

We have not been able to establish the global stability of $E_0^*$ when ${\mathcal {R}}_c < 1$. There are some vaccination models that exhibit the phenomenon of backward bifurcation, where a stable endemic equilibrium co-exists with the stable disease-free equilibrium Gumel (2002). However, based on the simulation results presented in the next section, we conjecture that Theorem 3.2 holds for the entire domain of system (19) and $E_0^*$ is globally stable.

#### Uniform persistence

In this section, we prove the disease persistence when ${\mathcal {R}}_c > 1$. Consider the semiflow ${\varPhi }(t)$ in ${\mathcal {X}}$, defined by the unique global solutions. We define the persistence function:

$$\begin{aligned} P: {\mathcal {X}}\rightarrow {\mathbb {R}}_{+}, \quad P(\phi )=\phi _8(0). \end{aligned}$$

Let

$$\begin{aligned} {\mathcal {X}}_{+}&:=\{\phi \in {\mathcal {X}}|P(\phi )>0\},\\ {\mathcal {X}}_{0}&:=\{\phi \in {\mathcal {X}}|P(\phi )=0\}= {\mathcal {X}}\setminus { {\mathcal {X}}_{+}}, \end{aligned}$$

where ${\mathcal {X}}_{0}$ is the extinction space corresponding to *P* (i.e., ${\mathcal {X}}_{0}$ is the collection of states without disease presence). From Theorem 3.1, it follows that ${\mathcal {X}}_{0}$ and ${\mathcal {X}}_{+}$ are forward invariant under the semiflow ${\varPhi }$.

##### Theorem 3.4

If ${\mathcal {R}}_c>1$, then the semiflow ${\varPhi }$ is uniformly *P*-persistent, i.e. there is a $\delta >0$ such that for any solution $\liminf _{t \rightarrow \infty } I(t)\ge \delta$.

##### Proof

First we show that $E^*_0$ is weakly *P*-repelling. Suppose that there exists $\psi _{0}\in {\mathcal {X}}$ such that $P (\psi _{0})>0$ with

24 $$\begin{aligned} \displaystyle \lim _{t\rightarrow \infty }{\varPhi }(t,\psi _{0})=E_0^*. \end{aligned}$$

For such a solution, $I(0)>0$ and $\lim _{t \rightarrow \infty } I(t)=0$. For sufficiently small $\epsilon >0$, we have ${\mathcal {R}}_c-\displaystyle \frac{6\epsilon }{\gamma + \mu }>1$. Hence, for sufficiently large *t*, we get $\Vert {\varPhi }(t,\psi _{0})-E_0^*\Vert <\epsilon$, and

$$\begin{aligned} \begin{aligned} I^\prime&\ge {\hat{\beta }}[S^\circ +V_s^\circ +\eta (V_p^\circ +V_d^\circ +V_w^\circ +W^\circ ) - 6 \epsilon ]I -(\gamma + \mu ) I \\&= \left[ {\mathcal {R}}_c - \frac{6\epsilon }{\gamma + \mu } - 1\right] (\gamma + \mu )I>0, \end{aligned} \end{aligned}$$

which contradicts the convergence of *I* to zero. Thus, $E_0^*$ is weakly *P*-repelling. We also note that whenever $I(t)\equiv 0$, from the *R* equation in (11) and Theorem 3.1, it follows that $R(t) \equiv 0$, $V_s \rightarrow V_s^\circ$, $V_p \rightarrow V_p^\circ$, $V_d \rightarrow V_d^\circ$, $V_w \rightarrow V_w^\circ$, $V_b \rightarrow V_b^\circ$, $W \rightarrow W^\circ$, and consequently $S \rightarrow S^\circ$ as $t\rightarrow \infty$. Therefore $\displaystyle \cup _{\phi \in {\mathcal {X}}_{0}}\omega (\phi )=\{E_0^*\}$, and one can see that ${\varPhi }$ is uniformly weakly *P*-persistent (Smith and Thieme 2011, Theorem 8.17). Since ${\varPhi }$ has a compact global attractor on ${\mathcal {X}}$, the application of Theorem 4.5 in Smith and Thieme (2011) guarantees that ${\varPhi }$ is uniformly *P*-persistent.

$$\square $$

##### Remark 3.5

It is expected that an endemic equilibrium exists when the disease is uniformly persistent. Due to the exponential terms in the model, it is not possible to derive an explicit formula for the components of an endemic equilibrium, and we could not prove the existence or uniqueness of such equilibrium using established methods (such as fixed point arguments). However, our numerical experiments suggest that there is a unique endemic equilibrium that emerges as ${\mathcal {R}}_c$ increases and passes the threshold of one. Regarding its stability, it is known that SIRS models with delay can exhibit periodic oscillations Hethcote et al. (1981), and their endemic equilibria can either be stable or unstable. For our model, a linear stability analysis seems very difficult to conduct due to the various delay terms. In numerical simulations, however, we can readily find a combination of parameter values for which the vaccination model (19) exhibits sustained oscillations in the disease prevalence. This typically occurs for a small vaccination coverage, and increasing this coverage first stabilizes the endemic equilibrium, and then can lead to disease elimination when it is sufficiently high to bring ${\mathcal {R}}_c$ less than one.

## Simulation results

To illustrate the effect of booster schedule on the dynamics of disease spread in the population, we simulated the model while varying the protection efficacy of primary vaccination and the coverage of booster vaccination. For the simulation results presented here, we used parameter values estimated for *Haemophilusinfluenzae* serotype b (Hib) in the published literature. Primary vaccination for Hib in most routine infant immunization programs includes 2 to 3 doses of vaccine offered between 2 to 6 months of age (World Health Organization et al. 2016), and we therefore assumed $\tau _s=6$ months for completion of primary series. Primary vaccination is estimated to provide partial protection for a fixed duration of $\tau _w=4$ years, followed by an exponentially distributed time period with the average of $1/\theta =6$ years (Konini and Moghadas 2015; Jackson et al. 2012). Booster vaccination provides full protection for a fixed period of $\tau _b=6$ years (Konini et al. 2016; Leino et al. 2000). We assumed that, after the period of full protection has elapsed, partial protection follows the same timelines as primary vaccination. Similar to booster vaccination, we assumed that recovery from infection provides full protection for a fixed period of $\tau _r=4$ years (Konini and Moghadas 2015). Infection in the form of carriage (i.e., asymptomatic without showing clinical symptoms) contributes more significantly to the incidence of Hib compared to symptomatic disease, and has a prolonged infectious period from several days to several weeks (Leino et al. 2000; Jackson et al. 2012). We therefore assumed an average infectious period of $1/\gamma =50$ days.

For the purpose of simulations, we used a population of size $N=100,000$ with an average lifetime of $1/\mu =70$ years. The transmission parameter $\beta$ was calculated based on a given basic reproduction number, while fixing other parameters of the model. We assumed ${\mathcal {R}}_0=1.2$ in the range 1.1–1.4 estimated in studies of Hib (Farrington et al. 2001) and other pathogens that cause bacterial meningitis, such as *Neisseria meningitidis* serotype C (Stephens 2011). We fixed the coverage of primary vaccine at $p=0.9$, and varied the protection efficacy of primary vaccination, reflected in the reduction of susceptibility to infection. We ran the simulations while changing the time for booster vaccination within the fixed period of partial protection following primary vaccination, that is, $0<\tau _p\le \tau _w=4$ years.

**Fig. 3:**
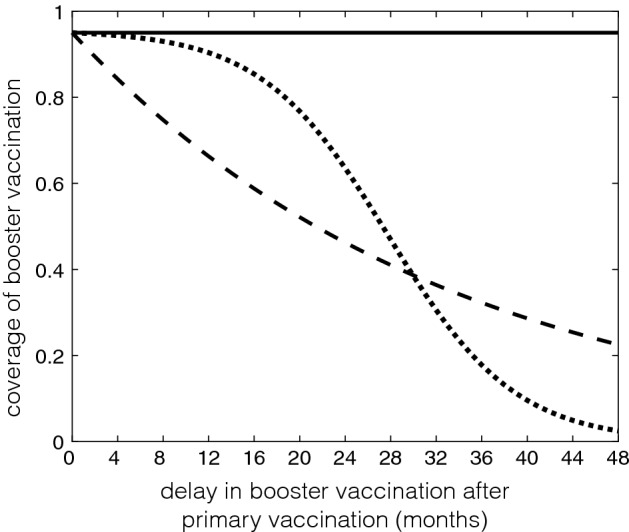
The coverage of booster vaccination ($\rho$) as a function of time delay ($\tau _p$) following primary vaccination. Solid line corresponds to a fixed coverage; dashed line represents the exponentially declining booster coverage; and dotted line illustrates an inverted logistic coverage of booster vaccination declining over time

We also simulated ${\mathcal {R}}_c$ as a function of two model parameters, namely the protection efficacy of primary vaccination ($\eta$), and the time for booster vaccination following primary series ($\tau _p$). For these simulations, we considered the coverage of booster vaccination as a function of $\tau _p$ in three different scenarios:

(i) Fixed coverage of booster vaccination: $\rho =0.95$ (Fig. 3, solid line).
(ii) Exponentially declining coverage of booster vaccination:
  $$\begin{aligned} \rho (\tau _p)=0.95e^{-0.001\tau _p}. \end{aligned}$$
  This coverage reduces as the time delay $\tau _p$ in booster vaccination increases (Fig. 3, dashed line).
(iii) Inverted logistic declining coverage of booster vaccination:
  $$\begin{aligned} \rho (\tau _p)=\displaystyle \frac{14.2307e^{-0.006\tau _p}}{0.1+e^{-0.006(\tau _p-450)}}. \end{aligned}$$
  This coverage reduces with time delay $\tau _p$ in booster vaccination in a functional form similar to van Genuchten-Gupta model (Fig. 3, dotted line).

**Fig. 4:**
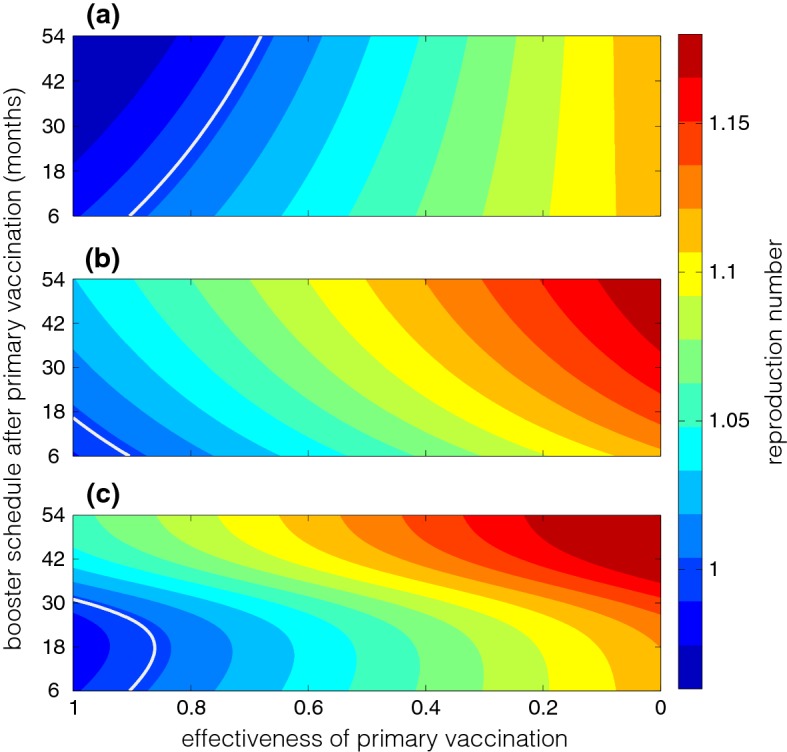
Reproduction number (${\mathcal {R}}_c$) as a function of protection efficacy of the primary vaccination ($1-\eta$, $x-$axis) and delay in the booster dose schedule ($\tau _p$, $y-$axis). The coverage of booster dose is: **a**$\rho =0.95$ fixed; **b**$\rho =0.95e^{-0.001\tau _p}$; and **c**$\rho =14.2307e^{-0.006\tau _p}/\big (0.1+e^{-0.006(\tau _p-450)}\big )$. The white curve corresponds to $R_c=1$

Figure 4 shows the variation in ${\mathcal {R}}_c$ corresponding to the scenarios of booster coverage. For a fixed coverage of booster vaccination ($\rho =0.95$), Fig. 4a shows that when the protection efficacy of primary vaccination is sufficiently high (approximately above 70%), ${\mathcal {R}}_c$ decreases with increasing delay in booster schedule following primary vaccination, and the disease can be eliminated if ${\mathcal {R}}_c<1$ (in the region to the left side of the white line). When primary vaccination provides a protection efficacy that is nearly as good as that conferred by the booster dose (i.e., $\eta >0.9$), the disease can be eliminated regardless of the time for booster schedule. However, for a moderate to low protection efficacy of primary vaccination, the delay in booster vaccination has little or no effect in reducing ${\mathcal {R}}_c$ and the disease persists in the population.

When the coverage of booster vaccination declines exponentially, we observed lower ${\mathcal {R}}_c$ for early booster schedule, regardless of the protection efficacy of primary vaccination (Fig. 4b). In our simulations, disease elimination can occur with a protection efficacy above 90%, but requires booster vaccination within 12 months following the primary vaccination (i.e., the region below the white line in Fig. 4b). In contrast to the scenario for a fixed coverage of booster vaccination, these simulations suggest that an early booster dose may be essential in curtailing disease spread if the coverage of booster vaccine is expected to decline (exponentially) over time. This scenario may correspond to vaccine refusal in the contexts of booster deferral (Centers for Disease Control and Prevention et al. 2009).

Further simulations indicate that functional form of the decline in booster coverage can also play an important role in determining the optimal timing of booster vaccination. With the inverted logistic functional form of $\rho$ represented by the dotted curve in Fig. 3, we observed that the protection efficacy of primary vaccination can influence the magnitude of reduction in ${\mathcal {R}}_c$ with the time delay in booster schedule (Fig. 4c). For a moderate to low protection efficacy of primary vaccination, early booster (similar to the scenario of exponential decline in $\rho$) leads to the maximum reduction in ${\mathcal {R}}_c$. However, as the protection efficacy of primary vaccination increases (approximately above 50% in these simulations), the maximum reduction of ${\mathcal {R}}_c$ corresponds to an intermediate time-interval for booster vaccination. Figure 4c indicates that an optimal timing of booster schedule may lead to disease elimination, while the disease can persist in the population if the booster dose is offered too early or too late following primary vaccination.

**Fig. 5:**
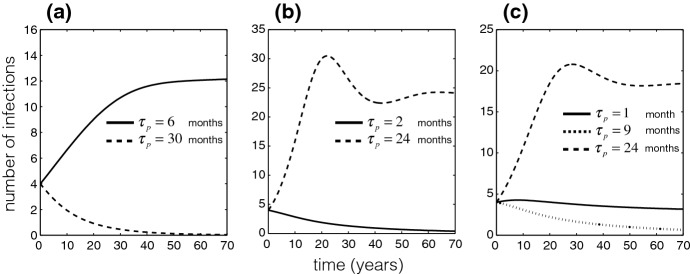
Prevalence of disease with: **a**$\eta =0.8$ and fixed $\rho =0.95$; **b**$\eta =0.95$, $\rho =0.89$ (for $\tau _p=2$ months) and $\rho =0.46$ (for $\tau _p=24$ months); **c**$\eta =0.88$, $\rho =0.95$ (for $\tau _p=1$ month), $\rho =0.92$ (for $\tau _p=9$ months), and $\rho =0.62$ (for $\tau _p=24$ months). The threshold of $\tau _p$ (for ${\mathcal {R}}_c=1$ in Fig. 4) is approximately (**a**) 19 months; **b** 7 months; and **c** 7 or 22 months

To further illustrate our findings in terms of disease prevalence, we simulated the model for the scenarios of booster coverage, while fixing the protection efficacy of the primary vaccination. Figure 5a shows that for fixed $\rho =0.95$ and $\eta =0.8$, the disease will be eliminated if the booster dose is offered 30 months after the primary vaccination. However, an earlier schedule of a booster dose 6 months after the primary vaccination leads to the disease persistence in the population. This situation is reversed for the scenario of booster coverage that declines exponentially with time delay in booster vaccination. Figure 5b shows the disease elimination and persistence for $\eta =0.95$, with the booster dose schedules of 2 and 24 months after the primary vaccination, respectively. When the booster coverage declines in a functional form similar to the inverted logistic function, Fig. 5c shows the disease persistence for early and late booster schedules of 1 and 24 months after the primary vaccination with $\eta =0.88$. However, for an intermediate delay of 9 months in booster vaccination, the disease is eliminated over time.

### Remark 4.1

**Fig. 6:**
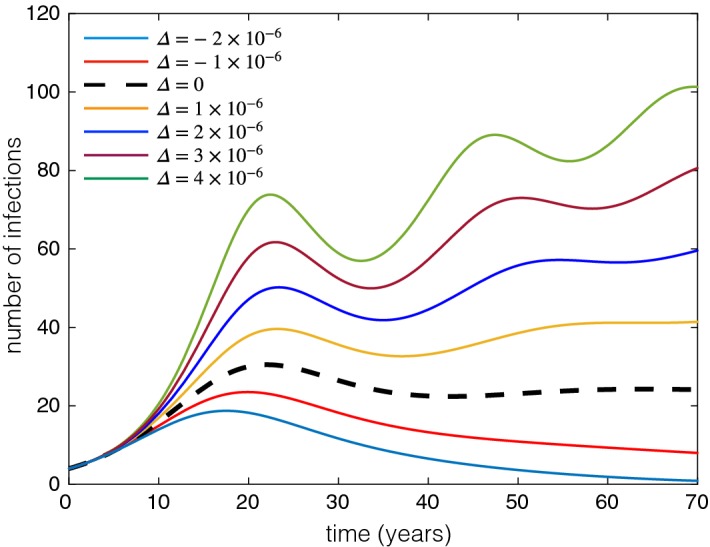
Prevalence of disease in varying populations. Dashed (black) curve corresponds to a constant population size (i.e., simulated dashed curve in Fig. 5b). Colour curves represent the disease prevalence over time with changing population size. Parameter values are the same as Fig. 5b with $\tau _p=24$ months, while the birth rate changes by ${\varDelta }$

For our theoretical results and simulations presented in Fig. 5, we assumed that the population size (*N*) is constant. To illustrate the effect of a changing population size on the disease dynamics, we considered the parameter setting of Fig. 5b with $\tau _p=24$ months, and modified the birth rate by some constant value ${\varDelta }$. Hence, ${\varDelta }>0$ corresponds to a growing population size, while ${\varDelta }<0$ represents a declining population. The results are illustrated in Fig. 6, showing the change in disease prevalence for different values of ${\varDelta }$ as the population size changes.

## Discussion

In this study, we investigated the role of booster schedule on the long-term disease dynamics. We developed a system of delay differential equations to include several key parameters describing the protection efficacy of primary vaccine series, durations of partial and full protection following vaccination, and coverage of primary and booster doses. In addition to investigating its dynamics, we simulated the model with a delay in booster dose after completing primary series using parameter values estimated for Hib. Simulation results indicate that, for a given protection efficacy of primary vaccination, the reduction of disease transmissibility, reflected in the reproduction number (${\mathcal {R}}_c$), depends critically on the timing of a booster dose. However, the coverage of booster vaccination remains a key parameter influencing the optimal timing of a booster dose. When the uptake of a booster dose is expected to remain high, a delay in booster vaccination (within the expected duration of protection induced by primary vaccine series) may be beneficial in reducing ${\mathcal {R}}_c$, and could lead to disease elimination for a sufficiently high protection efficacy of primary vaccination. This is particularly important if the booster dose provides only a relatively short period of full protection compared with the average lifetime. However, vaccination programs may contend with the possible drop-out and decrease in the coverage of booster vaccination, whether due to acquiring infection after receiving the primary series, or simply due to individuals voluntarily forgoing (e.g., refusal of) the booster dose (Omer et al. 2009; Dubé et al. 2013; Briere et al. 2014). In this case, our simulations illustrate that if the coverage of booster vaccination decreases over time, then the timing of a booster dose can be essential to achieve the greatest reduction of disease prevalence over time.

This study has important implications for public health vaccination policies. First, for routine infant immunization programs with high primary and booster coverages, deferral of a booster dose within the average duration of protection induced by the primary series may be beneficial. However, the protection efficacy of the primary vaccine series remains an important parameter in determining the optimal dosing interval between primary and booster vaccination (Charania and Moghadas 2016). Second, in the absence of efforts to achieve an optimal schedule, having a booster program does not necessarily guarantee the elimination of disease, even though the incidence may be reduced as has been observed for Hib. Given the high protection efficacy of primary vaccine series ($>85\%$) against Hib disease (Jackson et al. 2013), and weak evidence of additional protection from booster within one year following complete primary vaccine series, our results suggest that immunization programs should consider a longer time interval between primary and booster doses. Furthermore, the sensitivity of long-term disease outcomes to the booster schedule underscores the importance of targeted efforts towards improving uptake rates of both primary series and booster vaccination. It is also important to note that our results herein apply to vaccines that confer only a temporary protection. Conspicuously, for a vaccine that provides a long-term full protection comparable to the average life-time, the best outcomes are achieved with the shortest time interval between the primary and booster vaccination.

Our study has several limitations that merit further investigation. Our model is based on the assumption of homogeneous mixing in the population dynamics of disease spread. It is well documented that heterogeneities and contact patterns can influence vaccination dynamics at both the individual and population levels (Metcalf et al. 2015). We assumed a uniform protection efficacy of primary and booster vaccination without considering immunological characteristics of individuals that affect the within-host immune dynamics. Our simulation results are based on the assumption that an anti-Hib polysaccharide conjugate vaccine provides stronger immune protection due to effects of carrier protein on stimulation and proliferation of immune responses. This is an important consideration in the development of conjugate vaccines for T-cell independent pathogens (such as Hib) in order to enhance immunogenicity in infants and young children (Goldblatt 2000). We therefore assumed a shorter period of full protection following recovery from natural infection compared to booster vaccination. However, in older individuals with competent immune system, natural infection can also lead to the development of adaptive immune memory and therefore induce strong protection effects with timelines similar to those conferred by conjugate vaccines (Goldblatt 2000). These considerations can be included in advanced computational frameworks, such as agent-based modelling (Laskowski and Moghadas 2014; Shoukat et al. 2018), in order to evaluate the effect of individual level characteristics on the population dynamics of disease spread and control in the presence of vaccination. Despite these limitations, our study provides a theoretical foundation for future studies involving more detailed computational and quantitative models to help improve vaccination programs and booster schedules against vaccine-preventable diseases that require multiple vaccine doses.

## References

[CR1] Alexander M, Moghadas S, Rohani P, Summers A (2006). Modelling the effect of a booster vaccination on disease epidemiology. J Math Biol.

[CR2] Briere EC, Rubin L, Moro PL, Cohn A, Clark T, Messonnier N (2014). Prevention and control of haemophilus influenzae type b disease: recommendations of the advisory committee on immunization practices (acip). MMWR Recomm Rep.

[CR3] Centers for Disease Control and Prevention et al (2009) Invasive haemophilus influenzae type b disease in five young children–Minnesota. Ann Emerg Med 54(1):83–85 10.1016/j.annemergmed.2009.05.020 19541044

[CR4] Charania N, Moghadas SM (2016) Modelling the effects of booster dose vaccination schedules and recommendations for public health immunization programs: the case of haemophilus influenzae serotype b. International Journal of Public Health p. in review 10.1186/s12889-017-4714-9 PMC5598080 28903749

[CR5] Dubé E, Laberge C, Guay M, Bramadat P, Roy R, Bettinger JA (2013). Vaccine hesitancy: an overview. Hum Vaccines Immunother.

[CR6] Ehreth J (2003). The global value of vaccination. Vaccine.

[CR7] Farrington C, Kanaan M, Gay N (2001). Estimation of the basic reproduction number for infectious diseases from age-stratified serological survey data. J R Stat Soc Ser C Appl Stat.

[CR8] Fitzwater SP, Watt JP, Levine OS, Santosham M (2010). Haemophilus influenzae type b conjugate vaccines: considerations for vaccination schedules and implications for developing countries. Hum Vaccines.

[CR9] Goldblatt D (2000). Conjugate vaccines. Clin Exp Immunol.

[CR10] Gumel A (2002). Causes of backward bifurcations in some epidemiological models. J Math Anal Appl.

[CR11] Hale J (1977). Theory of functional differential equations.

[CR12] Hale J (1988). Asymptotic behavior of dissipative systems.

[CR13] Hethcote HW, Stech HW, van den Driessche P (1981). Nonlinear oscillations in epidemic models. SIAM J Appl Math.

[CR14] Jackson C, Mann A, Mangtani P, Fine P (2013). Effectiveness of haemophilus influenzae type b vaccines administered according to various schedules: systematic review and meta-analysis of observational data. Pediatr Infect Dis J.

[CR15] Jackson ML, Rose CE, Cohn A, Coronado F, Clark TA, Wenger JD, Bulkow L, Bruce MG, Messonnier NE, Hennessy TW (2012). Modeling insights into haemophilus influenzae type b disease, transmission, and vaccine programs. Emerg Infect Dis.

[CR16] Konini A, Moghadas SM (2015). Modelling the impact of vaccination on curtailing haemophilus influenzae serotype ‘a’. J Theor Biol.

[CR17] Konini A, Nix E, Ulanova M, Moghadas SM (2016). Dynamics of naturally acquired antibody against haemophilus influenzae type a capsular polysaccharide in a Canadian aboriginal population. Prev Med Rep.

[CR18] Laskowski M, Moghadas SM (2014) A general framework for agent–based modelling with applications to infectious disease dynamics. In: BIOMAT 2013, proceedings of the international symposium on mathematical and computational biology, vol 9. World Scientific, p 318

[CR19] Leino T, Auranen K, Mäkelä P, Käyhty H, Takala A (2000). Dynamics of natural immunity caused by subclinical infections, case study on haemophilus influenzae type b (hib). Epidemiol Infect.

[CR20] Low N, Redmond SM, Rutjes AW, Martínez-González NA, Egger M, di Nisio M, Scott P (2013). Comparing haemophilus influenzae type b conjugate vaccine schedules: a systematic review and meta-analysis of vaccine trials. Pediatr Infect Dis J.

[CR21] Metcalf CJE, Andreasen V, Bjørnstad ON, Eames K, Edmunds WJ, Funk S, Hollingsworth TD, Lessler J, Viboud C, Grenfell BT (2015). Seven challenges in modeling vaccine preventable diseases. Epidemics.

[CR22] Omer SB, Salmon DA, Orenstein WA, deHart MP, Halsey N (2009). Vaccine refusal, mandatory immunization, and the risks of vaccine-preventable diseases. N Engl J Med.

[CR23] Riolo MA, King AA, Rohani P (2013). Can vaccine legacy explain the british pertussis resurgence?. Vaccine.

[CR24] Riolo MA, Rohani P (2015). Combating pertussis resurgence: one booster vaccination schedule does not fit all. Proc Natl Acad Sci.

[CR25] Shoukat A, Van Exan R, Moghadas SM (2018). Cost-effectiveness of a potential vaccine candidate for haemophilus influenzae serotype ‘a’. Vaccine.

[CR26] Smith HL, Thieme HR (2011) Dynamical systems and population persistence. Graduate Studies in Mathematics, vol 118. American Mathematical Society, Providence, RI

[CR27] Stephens DS (2011). Protecting the herd: the remarkable effectiveness of the bacterial meningitis polysaccharide-protein conjugate vaccines in altering transmission dynamics. Trans Am Clin Climatol Assoc.

[CR28] World Health Organization, et al. (2016) Who recommendations for routine immunization-summary tables. WHO, Geneva

[CR29] Xu Z, Zhao XQ (2012). A vector-bias malaria model with incubation period and diffusion. Discrete Contin Dyn Syst Ser B.

